# Extraction and Quantification of Words Representing Degrees of Diseases: Combining the Fuzzy C-Means Method and Gaussian Membership

**DOI:** 10.2196/38677

**Published:** 2022-11-18

**Authors:** Feng Han, ZiHeng Zhang, Hongjian Zhang, Jun Nakaya, Kohsuke Kudo, Katsuhiko Ogasawara

**Affiliations:** 1 Graduate School of Medicine Hokkaido University Sapporo Japan; 2 Graduate School of Health Sciences Medical Management and Informatics Hokkaido University Sapporo Japan; 3 Division of Advanced Diagnostic Imaging Development Graduate School of Medicine Hokkaido University Sapporo Japan; 4 Department of Diagnostic Imaging Faculty of Medicine Hokkaido University Sapporo Japan

**Keywords:** medical text, fuzzy c-means, cluster, algorithm, machine learning, word quantification, fuzzification, Gauss, radiology, medical report, documentation, text mining, data mining, extraction, unstructured, free text, quantification, fuzzy, diagnosis, diagnostic, EHR, support system

## Abstract

**Background:**

Due to the development of medical data, a large amount of clinical data has been generated. These unstructured data contain substantial information. Extracting useful knowledge from this data and making scientific decisions for diagnosing and treating diseases have become increasingly necessary. Unstructured data, such as in the Marketplace for Medical Information in Intensive Care III (MIMIC-III) data set, contain several ambiguous words that demonstrate the subjectivity of doctors, such as descriptions of patient symptoms. These data could be used to further improve the accuracy of medical diagnostic system assessments. To the best of our knowledge, there is currently no method for extracting subjective words that express the extent of these symptoms (hereinafter, “degree words”).

**Objective:**

Therefore, we propose using the fuzzy c-means (FCM) method and Gaussian membership to quantify the degree words in the clinical medical data set MIMIC-III.

**Methods:**

First, we preprocessed the 381,091 radiology reports collected in MIMIC-III, and then we used the FCM method to extract degree words from unstructured text. Thereafter, we used the Gaussian membership method to quantify the extracted degree words, which transform the fuzzy words extracted from the medical text into computer-recognizable numbers.

**Results:**

The results showed that the digitization of ambiguous words in medical texts is feasible. The words representing each degree of each disease had a range of corresponding values. Examples of membership medians were 2.971 (atelectasis), 3.121 (pneumonia), 2.899 (pneumothorax), 3.051 (pulmonary edema), and 2.435 (pulmonary embolus). Additionally, all extracted words contained the same subjective words (low, high, etc), which allows for an objective evaluation method. Furthermore, we will verify the specific impact of the quantification results of ambiguous words such as symptom words and degree words on the use of medical texts in subsequent studies. These same ambiguous words may be used as a new set of feature values to represent the disorders.

**Conclusions:**

This study proposes an innovative method for handling subjective words. We used the FCM method to extract the subjective degree words in the English-interpreted report of the MIMIC-III and then used the Gaussian functions to quantify the subjective degree words. In this method, words containing subjectivity in unstructured texts can be automatically processed and transformed into numerical ranges by digital processing. It was concluded that the digitization of ambiguous words in medical texts is feasible.

## Introduction

Owing to the development of medical data, several electronic medical reports such as clinical records have been created, which provide a large amount of clinical data to the medical professional [1]. They have been shown to have capabilities such as contributing to health care knowledge discovery processes, for example, disease phenotyping and diagnosis [2,3], identification of new associations [4], development of disease surveillance systems [5], and health care monitoring systems [6]. Electronic medical reports are categorized into structured and free-text formats [7]. Where unstructured clinical notes contain rich subjective information [8-10]. A radiology report records a patient’s condition created by a health care professional, such as a doctor, and contains medical evaluation information [11]. Although, with the advent of natural language processing (a branch of artificial intelligence applicable to unstructured textual data), clinical text mining is increasingly used in various health domains, there is still relatively little text-based machine learning research available compared to the more common structured numerical data-based analysis [12].

The traditional method involves manual extraction of symptoms. Hyun et al [13] extracted symptoms by manually defining a dictionary of symptoms. Jagannatha and Yu [14,15] and Fodeh et al [16] manually labeled and extracted symptoms. Matheny et al [17] set rules to extract symptoms. Chen and Sarkar [7] and Tamang et al [18] used a combination of several of these methods to extract symptoms. The disadvantage of these methods is that they require considerable time and labor costs, and are error-prone [11].

Reátegui and Ratté [11] and Wu et al [19] extracted symptoms using the entity naming and extraction method. It improves the disadvantages of the manual extraction method by using a computer to automatically extract named entities from a large amount of text to extract symptoms, but unstructured data needs to be associated with entities to extract symptoms. Therefore, there is a risk of errors in modeling and naming entity relationships, which can affect the results.

De Silva et al [20] used the machine learning least absolute shrinkage and selection operator (LASSO) or ridge regularization method to extract symptoms from unstructured text (Marketplace for Medical Information in Intensive Care III [MIMIC-III]) and to predict mortality in patients with diabetes. Since LASSO uses a machine learning approach, it does not require association with entities and saves manual time and labor cost.

Notably, these methods were limited to the extraction of symptoms [11,14-17,21,22] and did not extract words that express the degrees of these symptoms (hereinafter, “degree words”). These degree words also contain information about doctors and patients if extracted from unstructured data that can provide a basis for judgment in medical diagnostic systems, and therefore, it is necessary to process these data to further improve the judgment accuracy of the medical diagnostic system [22]. However, unlike structured data, these degree terms include the subjectivity of doctors. They do not have a unified standard and cannot be processed directly by using a computer [21].

In 1965, Zadeh [23] of the University of California, Berkeley published the first paper on fuzzy theory, submitting for the first time the concept of fuzzy sets from the perspective of set theory to describe things with fuzziness and to describe things in everyday life by fuzzy logical reasoning, similar to the human thinking patterns and the probability theory proposed by Zadeh [24], describing the difference between randomness and probability, which is considered as the second milestone in the development of fuzzy mathematics. The emergence of fuzzy theory has provided a solid theoretical foundation and effective tools for the wide application of fuzzy mathematics in pattern recognition and other fields.

The concept of fuzzy theory emphasizes the use of fuzzy logic to describe things in real life and to make up for the shortcomings of classical logic (binary logic), which cannot describe things with unclear boundaries. Human natural language is vague in its presentation, and it is difficult to fully describe real-world problems using the dichotomy of “right or wrong” and “good or bad.” Therefore, fuzzy theory uses the definition of a fuzzy set to define and quantify the membership function (membership rank) of the degree to which an event belongs to this set, and solves different problems by quantifying the membership rank (membership value) [25].

Fuzzy c-means (FCM) is an unsupervised soft computing technology developed by Dunn [26] in 1973 and improved by Bezdek et al [27] in 1981. Unlike the hard clustering method, the soft clustering method uses fuzzy sets [23], which can solve the problem of text ambiguity better. In fuzzy sets, membership indicates the matching degree between the element and the set, with membership values ranging from 0 to 1. Furthermore, the concept of membership is extended in the FCM method, wherein the membership matrix represents the membership values of the elements in multiple clusters. FCM is one of the most commonly used methods to solve fuzzy problems. Compared with other clustering methods, it is more flexible and can represent the degree of data affiliation more appropriately [28]. The proposed method has two main advantages. First, it is unsupervised; therefore, labeled data is not required during training. Second, it can resolve subjective word ambiguity in fuzzy sets and medical texts such as a description of symptoms.

Therefore, we propose using the FCM and Gauss membership methods to quantify the subjective degree words in the English-interpreted report of the MIMIC-III data set.

## Methods

### Overview

**Figure 1:**
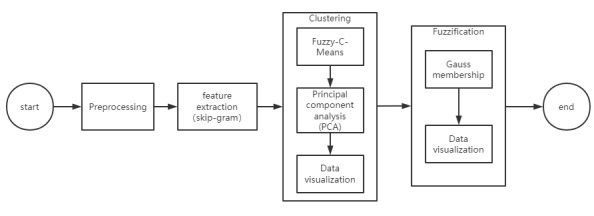
Overall structure of the proposed method.

The overall structure of the proposed method is shown in Figure 1. It contains five parts: preprocessing, feature extraction, clustering, digitization, and visualization. All the calculation methods used in this experiment were implemented in Python.

First, the raw data are preprocessed. Next, a skip-gram is used to extract word features and convert them into computer-processable data. Thereafter, the processed data are clustered. Subsequently, clustered data are digitized. Finally, the results of clustering and digitization are visualized to make the data structure easier to understand.

### Data Set

The data set used in this study was an interpretation report of 522,279 physicians from the MIMIC-III data set [29]. Five lung diseases (atelectasis, pneumonia, pneumothorax, pulmonary edema, and pulmonary embolus) were selected from Normalized Clinical Knowledge (NCK) [30] as keywords for the searched MIMIC-III data set. The search results are presented in Table 1. The number of texts used in the experiment was 381,091.

**Table 1:** Search results for 5 diseases.

Keywords	Texts (N=381,091), n
Atelectasis	119,765
Pneumonia	72,553
Pneumothorax	138,785
Pulmonary edema	47,364
Pulmonary embolus	2624

### Preprocessing

The input data set was preprocessed using normalization and Python code using the following tasks:

- Text classification: Filter text according to database keywords from NCK and classifications of various diseases
- Merge similar text: Merge all data for the same illness into one file to create a corpus of illnesses
- Remove special characters: All special characters (punctuation marks, question marks, exclamation marks, etc) are removed from the text and replaced with spaces
- Case exchange: Change all letters to lowercase to reduce vocabulary repetition
- Delete stop words: Delete stop words with no special meaning (eg, am, name)
- Tag words: To extract words, we used the Natural Language Toolkit to tag words.
- Word extraction: Degree words are primarily adjectives and adverbs, and illnesses are primarily nouns; therefore, only adjectives, adverbs, and nouns should be extracted.

### Feature Extraction

Feature extraction must be used to convert natural language into computer-processable numerical data. We used word2vec skip-gram [31] to extract features from the collected data. For the word vector to reflect the contextual relationships, the data used in the word vector training were not the extracted words but the text with the stop words removed. The extracted words were used for cluster analysis. Furthermore, to exclude irrelevant words, we removed words with fewer than 10 occurrences. The dimension of the word vector was set to 100 [32]. This implies that each word was represented as a 1 × 100 vector. Text consisting of N words was represented as a matrix size of N × 100. These word vectors can contain the positional relationship and structural information of each word in the text.

### Fuzzy C-Means

We used the FCM method to cluster the features. This method allows symptoms of the same disease in different texts to be grouped into the same category. Each element has a membership value for each category. The degree of membership depends on the distance from the element to the center of the cluster, and they are inversely proportional [33]. In this study, the number of clusters for each disease was set to 10. The fuzzy index m setting was set to 2 [34]. The loop was set to 50 times.

### Fuzzification

To import the trained model as a basis for evaluation into a medical diagnostic system, digitization must be used to convert the FCM results into a numerical range. In this experiment, the Euclidean distance from each word to its center was used as a reference to digitize the word. The formula for calculating the distance is shown in equation 1.



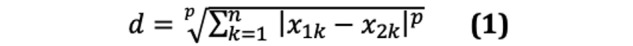



The more popular membership functions are the triangular membership function and the Gaussian membership function, and since the center of the triangular affiliation function is too steep, this experiment uses the Gaussian membership function for the numerical range conversion. This membership function is given by equation 2. The center of each Gaussian membership function can be determined based on these distances, where *x*_1_*_k_* is the *k*th coordinate value of the center and *x*_2_*_k_* is the *k*th coordinate value of the element.



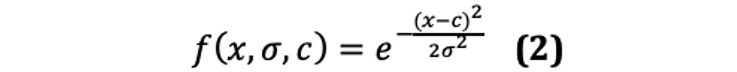



Where σ is the width parameter to control radial range of function. For clustering problems, if the clustering structure in the feature space of sample points is compact, the smaller σ can ensure the effect of the sample points clustering. If the clustering structure is dispersed, the larger σ can help to obtain the explicit membership function distribution. We set the σ value to 0.4.

It is necessary to ensure that the membership function takes values in the range of 0-1 in the fuzzy set [35]. Therefore, we set the boundary c to 0.1.

### Principal Component Analysis and Data Visualization

**Figure 2:**
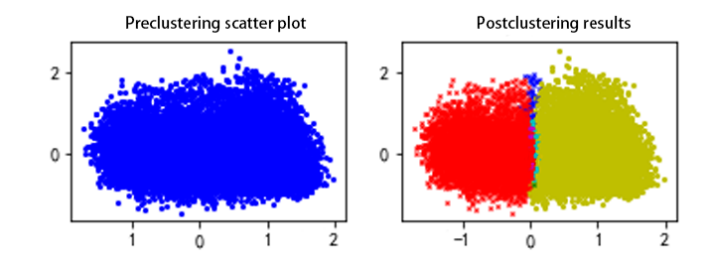
Visualization result of the disease atelectasis.

In addition, it is necessary to visualize the results of the two parts (ie, clustering and digitization). To visualize the results after clustering, principal component analysis (PCA) must be used to reduce the dimensions of the data. To make the data easier to understand, we used PCA [36] to project high-dimensional data into a low-dimensional space. PCA can reduce the dimensions of data while retaining key information and increasing readability. The numerical results will be transformed into easy-to-understand 2D plots. Figure 2 is the visualization result of the disease atelectasis.

## Results

This section introduces the results of this study. First, the results of the clustering process, data dimension reduction, and visualization are described. Thereafter, the results of the proposed fuzzy method are presented.

### Fuzzy C-Means

Textbox 1 lists the results of clustering different diseases. The [Atelectasis] cluster contained degree words such as “quickly” and “with small,” and symptom words such as “obstruction.” The [Pneumonia] cluster contained degree words such as “small” and “mid,” site words such as “pulmonary,” and symptom words such as “effusions.” The [Pneumothorax] cluster contained symptom words such as “pain” and “fever,” and degree words such as “expansion.” The [Pulmonary edema] cluster contained symptom words such as “nausea” and “neutropenic,” and degree words such as “copious.” The [Pulmonary embolus] cluster contained degree words such as “expansion.” Different diseases will produce different degree words and symptoms. These degree words and symptoms were used to judge diseases. To facilitate understanding of the variability of degree words between diseases, we have chosen the same words for explanation below.

**Clustering results. The italics indicate extracted degree words that are processed through fuzzification afterward.**

**Atelectasis**

“quickly,” “over penetrated,” “stenosis,” “*small*,” “qualified,” “midthigh,” “unlike,” “hepatic,” “wet massive,” “hypodensities,” “effusion with,” “*high*,” “obstruction,” “ultra-fast,” “tine”...

**Pneumonia**

“pleural,” “lung,” “*high*,” “effusion,” “pulmonary,” “tube,” “lobe,” “lower,” “contrast,” “atelectasis,” “unchanged,” “*small*,” “upper,” “bilateral,” “normal,” “edema,” “effusions,” “opacity,” “within,” “consolidation,” “stable,” “mild,” “increased,” “consistent,”　“unremarkable,” “clear,” “moderate,” “enlarged,” “large,” “low,” “opacification,” “well,” “mid,” “slightly,” “improved,” “significant,” “improvement,” “severe,” “minimal,” “slight,” “decreased,” “less”...

**Pneumothorax**

“pain,” “fever,” “worsening,” “cough,” “increasing,” “expansion,” “exacerbation,” “vomiting,” “*small*,” “sputum,” “febrile,” “*high*,” “syncope,” “decreasing,” “overdose”...

**Pulmonary edema**

“swelling,” “*high*,” “nausea,” “diarrhea,” “rising,” “neutropenic,” “prematurely,” “copious,” “malaise,” “instability,” “small”...

**Pulmonary embolus**

“thin,” “reduced,” “*small*,” “inflated,” “expansion,” “*high*,” “anomaly,” “hyperostosis,” “overinflation,” “heights”...

### Fuzzification

**Figure 3:**
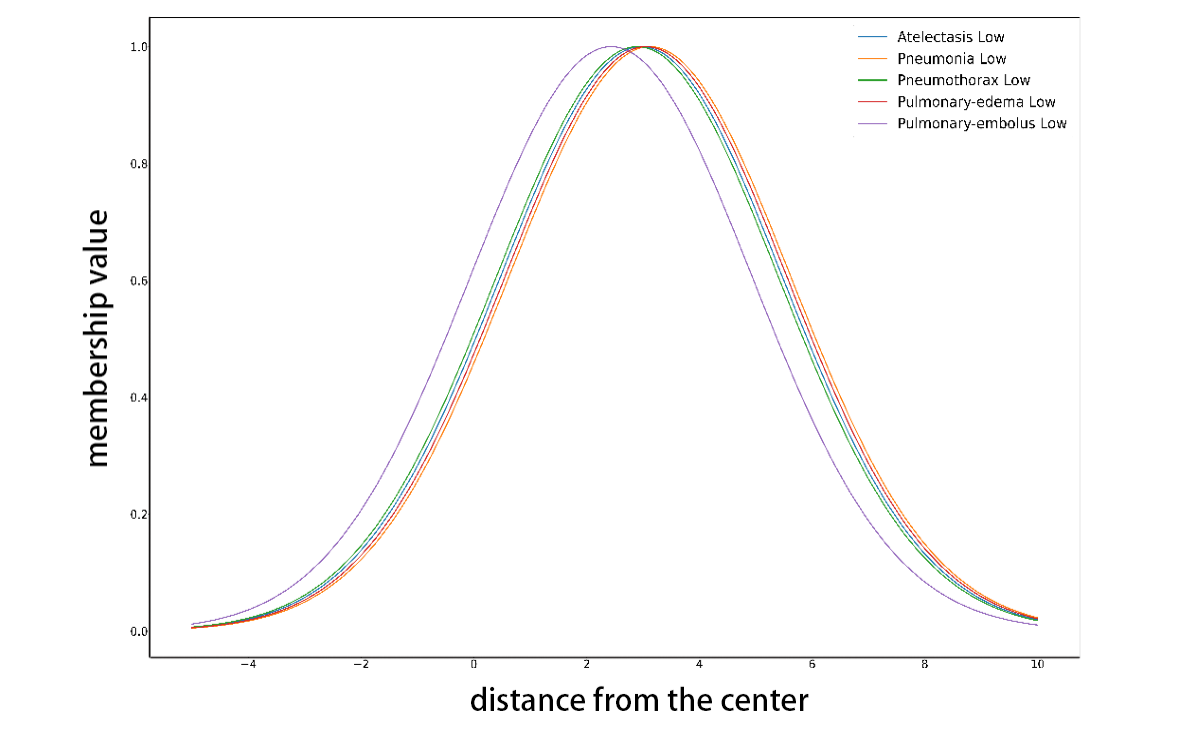
Membership chart of the degree word low.

Figure 3 shows a membership diagram of the degree word “low.” The x-axis represents the distance from the center. The y-axis represents the membership value. The median Gaussian membership is the distance from this element to the cluster’s center. The greater the distance from the center point, the lower the degree of membership. The maximum value at the center point is 1.

Table 2 shows the central values for membership of the same word for the five illnesses. The differences between conditions can be determined by comparing the same vocabulary for different conditions. By comparing the words of each degree, we can identify the presenting characteristics of each symptom and create a table of the possible symptoms of the patient by comparing them to the patient’s presenting characteristics.

**Table 2:** Medians of the same word for 5 illnesses.

	Low, median	High, median	Small, median	Large, median
Atelectasis	2.9713	2.7161	2.4547	2.4047
Pneumonia	3.1217	2.9383	2.4354	2.4190
Pneumothorax	2.8998	2.8240	2.3956	2.3285
Pulmonary edema	3.0518	2.9880	2.4539	2.4003
Pulmonary embolus	2.4357	1.7908	2.6419	2.5570

## Discussion

### Principal Findings

Our proposed method is capable of identifying and extracting degree words from unstructured text (Textbox 1) and transform them into computer-recognizable membership values (Table 2). The results in Textbox 1 show that all extracted words contain the same subjective words (low, high, etc), which allows for an objective evaluation method. These same ambiguous words can be used as a new set of feature values to represent the disorders. That is, the same subjective words can be used to determine a disease. Thereafter, we quantified the extracted subjective words using the Gaussian membership method to transform the features contained in the ambiguous words into numerical features.

For the fuzzification section, we used the Gaussian fuzzy function to transform the fuzzy words extracted from the medical text into numbers. The results in Figure 3 and Table 2 show that it is feasible to transform the features contained in the subjective words into digital features. The digitization of ambiguous words (symptomatology and degree words) can improve the use of medical texts, which is crucial for improving the accuracy and interpretability of medical diagnostic systems. Furthermore, we will verify the specific impact of the quantification results for ambiguous words such as symptom words and degree words on the use of medical texts in subsequent studies to improve the accuracy and interpretability of medical diagnosis systems.

### Comparison With Other Methods

Fodeh et al [16] used a random forest classifier to identify clinical notes with pain assessment information. They extracted subjective words describing pain intensity such as mild, moderate, and severe from the EHR and used them to help determine whether the text described pain. The results showed higher accuracy for the method using subjective words. The conclusion that subjective words can be used to further improve the medical diagnostic system is the same as in this study. Unlike this study, they only classified the text by subjective words and did not further use subjective words as a basis for a new condition determination. This study translates the subjective words into features of the disorder by fuzzification.

Matheny et al [17] pointed out the importance of symptoms and proposed a rule-based approach to extract them. This method is compared to this study, which requires using detailed rules, the creation of which is limited to the medical personnel. Manually writing rules requires considerable time and may not be exhaustive. The unsupervised method in this study requires no manual processing and no human involvement, reducing time consumption and extraction omissions. Additionally, while they note the importance of symptoms for the disorder, they do not extract and analyze the subjective words.

The method that uses a dictionary to extract symptoms also requires considerable time to construct and is not general [37]. For different tasks, rules need to be adapted. Additionally, the rule formulation process involves deleting and adding rules and discussing the rule coverage. Therefore, substantial time cost is required. Whereas, the FCM method is not limited to a specific scope and has excellent generality. The rules are summarized and extracted from the text, so they are more broadly applicable.

De Silva et al [20] used the LASSO method to predict mortality for patients with diabetes in MIMIC-III. The prediction results suggest that the clinical text provides a resource for future optimization and personalization of diabetes care. Additionally, the LASSO method has strong generalizability. However, a large amount of data is required to obtain more accurate results, but currently there are far less data with fine-grained labels than unlabeled data in the medical field. This method is equally effective for unlabeled data since it is an unsupervised method, but the impact on the accuracy of the results needs further discussion.

Reátegui and Ratté [11] used a named entity extraction system to extract symptoms. It extracts symptoms by automatically extracting named entities from large amounts of text using a computer, but it can create errors in modeling and named entity relationships that can affect the results. As the methods in this study do not need to be bound to named entities, these errors do not occur.

Wu et al [19] developed a deep neural network (DNN) to generate word vectors from a large, unlabeled, Chinese corpus by unsupervised learning. The results show that the DNN-based approach can capture grammatical features by word vectors and achieves higher performance compared with the traditional conditional random fields approach. The time spent will be substantially reduced by unsupervised learning. It is noted that further performance improvement will be achieved if high-dimensional discrete features are used. However, it is difficult to use DNNs as a basis for diagnostic systems because of their black box effect. However, this study is able to give the basis (affiliation) of the results while outputting the results through the affiliation function. Therefore, it can be used as a reference for medical practitioners.

### Limitations and Further Study

This study has some limitations. The first is data. There were abbreviations and misspellings in the data, which were not extracted. The study has confirmed the presence of a large number of abbreviations in unstructured texts that contain equally large amounts of information [17]. We will try to extract and analyze the presence of abbreviated words in the data in a subsequent study. Additionally, the word2vec method we used has the limitation that the vector representation does not change when the words are used in different contexts. Due to the different clinical contexts of diseases, a comparative study using context-sensitive embedding algorithms is also needed. Finally, the results for each word calculated in this experiment need to be verified by the medical diagnostic system, and the method we propose will be verified in future studies.

This study is mainly about the training of the model and discusses whether it is possible to quantify the subjective word and use it as an indicator to determine the disease. In this study, we used data from different conditions to quantify the subjective words contained in them separately. Concerning future research, we envision using the quantified data to process hospital-specific data to give accuracy rates. For clinical application, we will perform disease prediction based on the subjective words in the data to get a list of possible diseases of the patient and give the correlation rate based on the fuzzy graph (Figure 3) generated in this study. Thus, it will assist physicians in diagnosis. Additionally, this method can solve the black box problem.

### Conclusion

This study proposes an innovative method for handling subjective words. We used the FCM method to extract the subjective degree words in the English-interpreted report of MIMIC-III and then used the Gaussian functions to quantify the subjective degree words. In this method, words containing subjectivity in unstructured text can be automatically processed and transformed into numerical ranges by digital processing. The results show that membership medians of “low” were 2.971 (atelectasis), 3.121 (pneumonia), 2.899 (pneumothorax), 3.051 (pulmonary edema), and 2.435 (pulmonary embolus). This proves that the digitization of ambiguous words in medical texts is feasible. Our results proved the feasibility of using subjective words in medical texts. This makes it possible for medical researchers to further improve the use of electronic medical texts, thus affecting the accuracy and interpretability of the medical diagnostic system. In future research, we will consider how to extract abbreviations from abbreviation dictionaries and texts, as well as further validate the impact on the accuracy and interpretability of the medical diagnostic system.

## Abbreviations

DNN: deep neural network
FCM: fuzzy c-means
LASSO: least absolute shrinkage and selection operator
MIMIC-III: Marketplace for Medical Information in Intensive Care III
NCK: Normalized Clinical Knowledge
PCA: principal component analysis
